# Deep learning for discriminating cochlear malformations on temporal bone CT

**DOI:** 10.1016/j.bjorl.2026.101811

**Published:** 2026-04-28

**Authors:** Zhenhua Li, Langtao Zhou, Xiang Bin, Bin Liu, Anzhou Tang, Songhua Tan

**Affiliations:** a The First Affiliated Hospital of Hunan Normal University, Hunan Provincial People's Hospital, Department of Otorhinolaryngology‐Head and Neck Surgery, Changsha, Hunan, China; b Beijing Institute of Technology, The School of Optics and Photonics, Beijing, China; c The First Affiliated Hospital of Guangxi Medical University, Department of Otorhinolaryngology Head and Neck Surgery, Nanning, Guangxi, China

**Keywords:** Cochlear malformation, Deep learning, Temporal bone, Cochlear implant

## Abstract

**Objectives:**

Diagnosis of cochlear malformation on temporal bone CT images is often difficult because the imaging findings are frequently subtle. Our aim was to assess the utility of deep learning analysis in diagnosing cochlear malformation on temporal bone CT images.

**Methods:**

A total of 373 temporal bone CTs (187 with normal and 186 with malformed cochlea) were included in this study. The network Swin UNETR was used for cochlea automatic segmentation, then we divided it into left and right sides. three classification networks, ResNet50, EfficientNet-B0 and DenseNet121, were used for training to diagnose cochlear malformation (590 sides). The test set (149 sides) was analyzed using deep learning models and by otologists.

**Results:**

The average Dice coefficient of Swin UNETR was 0.93. The AUCs for ResNet50, EfficientNet-B0 and DenseNet121 were 0.93, 0.89 and 0.93, respectively, with DenseNet121 achieving the highest AUC among the four models. ResNet50 and DenseNet121 are better than otologists (1‒5 y).

**Conclusion:**

The deep learning technique may be a useful supportive tool for diagnosing cochlear malformation on temporal bone CT images.

## Highlights

• Deep learning shows great promise for diagnosing cochlear malformation.
• ResNet50 and DenseNet121 demonstrate well compared to the chief physician.
• Deep learning may be a valuable aid for experienced radiologists.

## Introduction

The structure of the inner ear is complex, and even in normal people, there are large individual differences in the anatomy of the cochlea.^1^^,^^2^ Research data show that in normal-hearing people, the difference in the length of the largest and smallest cochlear tube can be as high as 40%.^3^^,^^4^ According to radiologic studies, it is estimated that 20% of patients with congenital sensorineural hearing loss have a malformed inner ear.^5^ Cochlear Implantation (CI) is one of the most important treatments for severe or profound sensorineural hearing loss. Preoperative images of the temporal bone should be fully evaluated, and the morphology of the cochlea should be carefully examined to evaluate the difficulty of surgery and judge the prognosis. When patients with cochlear malformations undergo cochlear implant surgery, the risk of complications increases.^6^ Furthermore, different electrodes can be selected according to different cochlear malformations.^7^

At present, the diagnosis of cochlear malformation is mainly based on CT scans of the temporal bone. The diagnosis of cochlear malformations was determined by referring to the classification of inner ear malformations proposed by Sennaroglu et al.^8^ in 2017. Due to the subtle and complex structure of the inner ear, studies have found that approximately one-third of milder malformations are clinically missed.^9^ Some subtle deformities are difficult to diagnose by CT of the temporal bone. Some studies have improved the diagnosis rate of cochlear malformations by using postprocessing techniques such as Multiplanar Reformation (MPR) in CT of the temporal bone.^10^ However, it is time-consuming and has certain limitations.

Recently, Computer-Aided Diagnosis (CAD) systems have become a crucial part of routine clinical work for detecting abnormalities on medical images. According to the imaging characteristics of different lesions, different DL algorithms are used for processing, which can improve the accuracy and timeliness of image diagnosis and reduce the work intensity of doctors. At present, the classic network designs in image classification include VGG,^11^ Inception v3,^12^ ResNet,^13^ etc. Generally, CAD systems include multiple steps, such as raw image acquisition, preprocessing, segmentation, feature extraction, and disease classification.^14^ Among the above steps, segmentation is a vital component since features for the classification can be obtained from the region of interest in the segmented mask. Due to the intricate anatomical structures within the temporal bone, the application of artificial intelligence is relatively limited. Up to now, it is used for the segmentation of the inner ear,^15^ the diagnosis of middle ear cholesteatoma,^16^ etc. In our previous research, we utilized deep learning methods to segment the cochlea^17^ and diagnose cochlear malformations in 2D imaging pictures.^18^ This article represented the first study to apply deep learning in the diagnosis of cochlear malformation based on 3D CT images.

The aim of our study was to assess the utility of deep learning methodology in diagnosing cochlear deformities on 3D temporal bone CT images.

## Methods

### Patients

**Fig. 1:**
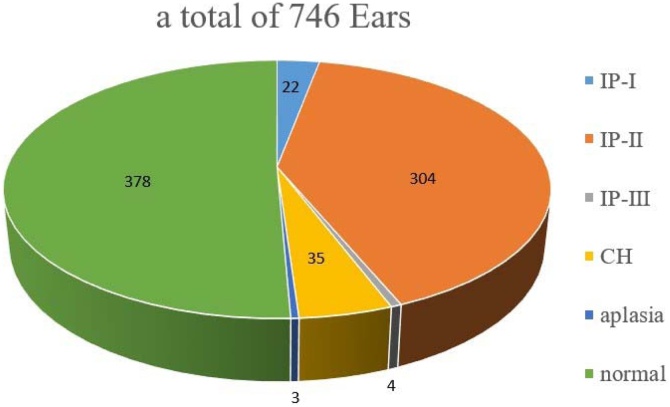
The morphological classifications of this study.

A total of 373 patients who underwent temporal bone CT examination from July 1, 2013, to November 30, 2021, were enrolled, including 187 patients with normal cochleae and 186 patients with deformed cochleae. Patients aged 2–90 years with a median age of 14-years were included. CT images of malformed cochlea showed that 3 sides of the cochlea showed aplasia and that 4-sides were absent after cochlear implantation. A total of 365 sides of malformed cochlea and 374 sides of normal cochlea were included (Fig. 1).

### CT imaging protocol

The scanning parameters were as follows: the tube voltage was 120/140 kV; the tube current was 140–300 mas; the matrix size was 512 × 512; the slice thickness was 0.625 mm/0.6 mm thick; the pitch was 0.32 mm; and the scan range was from the apex of the mastoid process to the upper edge of the orbit.

### Determination of the final diagnosis

The diagnosis of cochlear malformations was determined by referring to the classification of inner ear malformations proposed by Sennaroglu et al.^8^ in 2017. The gold standard is the unanimous diagnosis of three Associate chief otologists. When two of the three doctors disagree with the other one, the diagnosis concluded by the two agreeing doctors is the final diagnosis.

### Image analysis

#### Image selection for deep learning analysis

First, manual segmentation of the cochlea was performed by an associate chief otologist with extensive experience in temporal bone CT scanning using 3D Slicer software. Then, all images are normalized to [0.1], and random flipping, rotation and intensity transformation (0.9–1.1) operations are used to enhance the data of images in the training set with a probability of 0.1.

#### Deep learning analysis

The workflow applied in this study consisted of two steps and is illustrated in Fig. 1. First, we used the segmentation network Swin UNETR^19^ for automatic segmentation of the cochlea, and then we divided the partitioned cochlea into left and right sides. 7 of the samples were excluded for postoperation or aplasia, resulting in a total of 739 sides. 149 ears (20%) were randomly selected as the test set, and the remaining 590 sides (80%) formed the training set. The three classification networks ResNet50,^20^ EfficientNet-B0^21^ and DenseNet121^22^ were used for the diagnosis of cochlear malformation.

### Training and validation

Experimental environment: CPU: Intel(R) Xeon(R) Silver 4310 CPU@ 2.10 GHz memory: 256 GB, graphics card: NVIDIA GeForce RTX 3090.

Hyperparameters and models: 1) Swin UNETR: The number of epochs was 400, and the batch size = 1. The resolution of the patch used in this study was 128 × 128 × 32. Using the AdamW optimizer, cross-entropy and Dice loss were used to train the model. The initial learning rate was 0.0001, and the learning rate decay method was used to iteratively train the model, mainly including the cosine learning rate and warmup step decay, with warmup epoch = 20. 2) Automatic segmentation network: The number of epochs was 100, the AdamW optimizer was used, the initial learning rate was 0.0001, and cross-entropy and Dice loss were used to train the model. The resolution of the patch was 128 × 128 × 32, and the batch size was 4.

**Fig. 2:**
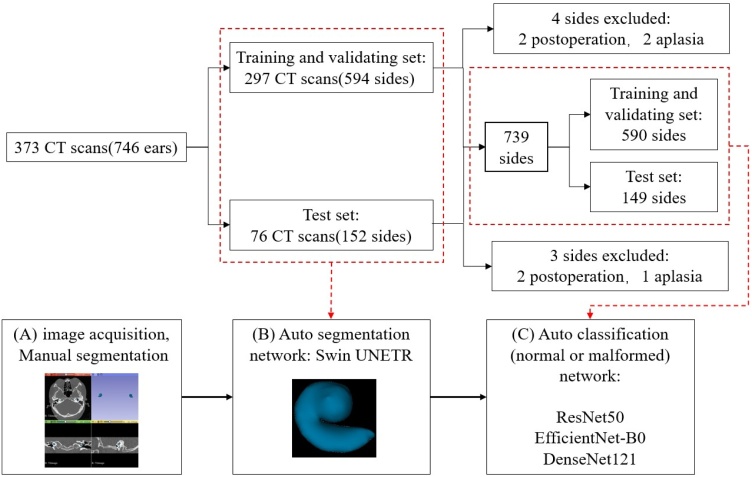
Schematic diagram illustrating the data processing and the workflow.

Subsequently, the dataset was randomly assigned as the training set (297 cases) and the test set (76 cases) at a ratio of 8:2 (Fig. 2). We did not distinguish the left and right sides in the automatic segmentation. In automatic diagnosis training, the left and right sides were judged normal or malformed respectively, among which 3 cochlear were missing and 4 cochlear were removed after surgery. In the classification diagnosis, 590 cochlear were used for training, and 149 were tested.

### Statistical analysis

The performance of the model was assessed by the Area Under the Curve (AUC) of the Receiver Operating Characteristic (ROC) curve reflecting the sensitivity and specificity of model predictions. The True Positive Rate (TPR), True Negative Rate (TNR), Positive Predictive Value (PPV), and Negative Predictive Value (NPV) were also calculated.

### Evaluation of expert performance

In this study, 4 otologists from the department of otorhinolaryngology head and neck surgery were recruited to evaluate their diagnostic performance on the test data sets dataset. They were blinded to the final diagnosis and the results of the deep learning analysis. they reviewed all temporal bone CT images (full-sized CT images in both axial and coronal planes) in the test cohort using 3D Slicer, with freely scrollable images and adjustable window level/width, and then identified these 149 temporal bone CT images as either having cochlear malformations or negative.

## Results

**Fig. 3:**
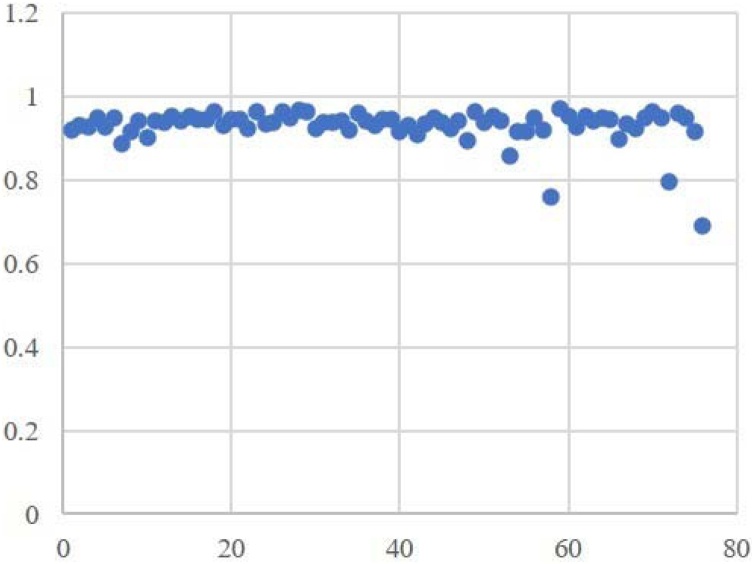
DSC of segmentation in test set.

**Fig. 4:**
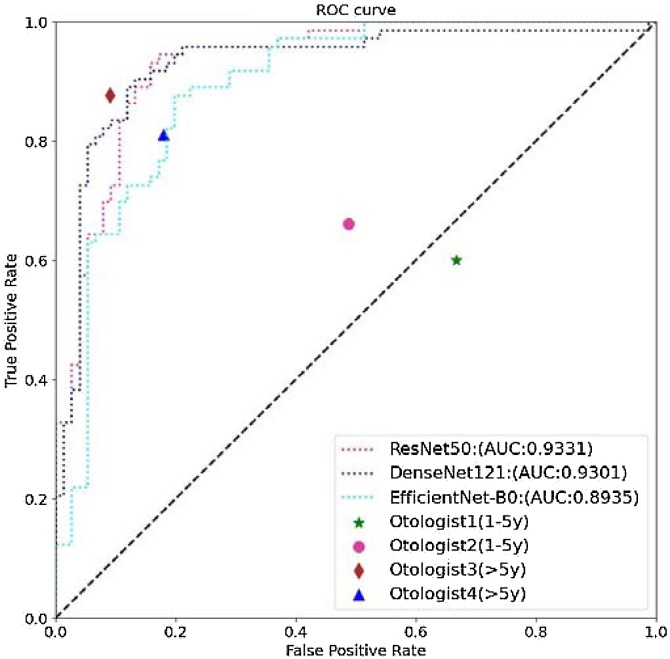
Results of ROC curve analysis. ROC curves for the deep learning models of ResNet10, ResNet50, SE-ResNet50 and DenseNet121 are shown.

Swin UNETR performed automatic segmentation, and the average Dice coefficient was 0.93 (Fig. 3); the highest was 0.97, and the lowest was 0.76. The cochleae obtained by automatic segmentation were then trained in the three classification network models ResNet50, EfficientNet-B0 and DenseNet121. The test set results are shown in the table below (Table 1). The AUCs for ResNet50, EfficientNet-B0 and DenseNet121 were 0.93, 0.89 and 0.93 (Fig. 4), respectively, with ResNet50 achieving the highest AUC among the four models. The best accuracy rate of the otologists for cochlear malformation was ACC 0.86.

**Table 1:** Diagnostic performance of DL models on a separate test dataset.

	PPV	NPV	TPR	TNR	ACC
ResNet50	0.91	0.77	0.71	0.93	0.82
DenseNet121	0.94	0.75	0.67	0.96	0.92
EfficientNet-B0	0.81	0.78	0.75	0.83	0.79

## Discussion

In this paper, deep learning technology was applied to CT images of the temporal bone for the first time to aid in the 3D diagnosis of cochlear deformity, and satisfactory results were obtained. The performance of the DL models ResNet50 and DenseNet121 was as high as that of the otologists (>5 y).

Previous studies have shown that abnormalities in the bony inner ear structure can be found on CT images in approximately one-quarter of patients with congenital deafness. With the diversification of cochlear implant electrode design, the preservation of residual hearing and personalized customization of electrodes have become the focus of current CI surgery.^23^ It is necessary to determine whether there is cochlear malformation before surgery, which can help surgeons select the length and type of electrodes suitable for patients before surgery, predict the difficulty of surgery, minimize the degree of damage to the cochlear structure, and achieve the purpose of preserving residual hearing.^24^ In classifying cochlear malformations, due to multiple factors affecting CT images such as CT scan parameter setting, image quality, and clinician experience, some minor cochlear malformations are not easy to diagnose, especially for inexperienced ear doctors with limited experience or otolaryngology head and neck surgeons without subspecialty training. The judgment of cochlear malformations is very subjective, and the diagnosis rate is low.

With the development of deep learning, many scholars have applied it to temporal bone CT for the automatic diagnosis of ear diseases or lesions. In 2021, Fujima et al.^25^ conducted CT of temporal bone through four kinds of network models for automatic diagnosis of otosclerosis. The study was divided into two steps: first, 2D rectangular box target detection was conducted in the vestibular window area, and then classification diagnosis was determined. Wang et al.^26^ used Inception-V3 to classify 562 patients with normal, chronic suppurative otitis media and middle ear cholesteroma. Eroglu et al.^27^ used three deep learning network models and SVM to classify and diagnose chronic suppurative otitis media and cholesteatoma otitis media via CT of the temporal bone. Takahashi et al.^28^ used MobileNetV2, a relatively lightweight neural network model, to train and predict CT images of the temporal bone of 164 patients to determine whether cholestatoma extends to the mastoid. All the above studies are based on the two-dimensional diagnosis of single or multiple temporal bone CT scans. However, in practice, otologists or radiologists tend to conduct comprehensive analyses based on both the upper and lower levels and make diagnoses based on single or multiple 2D images, which has great limitations.

So far, no other scholar has diagnosed cochlear malformations through artificial intelligence methods. In our previous research,^18^ we utilized deep learning methods to diagnose cochlear malformations in 2D imaging pictures. it was only limited to one two-dimensional CT image.it can’t provide an entire coverage of the cochlear region. Therefore, we hope to improve our research. This article represented the first study to apply deep learning in the diagnosis of cochlear malformation based on 3D CT images. In this study, the cochlea was automatically segmented first and then classified to determine whether there was malformed cochlea. The ResNet50, DenseNet121 and DenseNet121 models all achieved good results. Swin UNETR is a U-shaped network architecture that uses Swin Transformer as an encoder, using a CNN-based decoder that connects to the encoder via skip connections of different resolutions. This is the first transformer-based pretraining framework designed specifically for self-supervised tasks in 3D medical image analysis. In computed tomography, Convolutional Neural Networks (CNNs) have become the most mainstream methods, such as GoogLeNet, VGG-19, Inception and other models. A milestone event in the history of CNN was the emergence of the ResNet model, which can train deeper CNN models to achieve greater accuracy. The core of ResNet's model is to train a deeper network by establishing shortcuts (skip connections) between the front layer and the back layer, which facilitates the backpropagation of gradients during training. DenseNet is mainly based on the idea of ResNet shortcuts. The difference is that it adopts a denser connection mode, which is a dense convolutional neural network, and the former propagated mode densely connects each layer with the other layers. The purpose of this is to ensure the maximum flow of information between layers and to connect all layers (feature map size matching) directly together. Compared with ResNet, DenseNet better preserves the gradient to avoid its disappearance, enhances feature propagation among networks, realizes and strengthens feature reuse, effectively reduces the number of parameters and obtains increasingly better results through continuous optimization of gradient flow. Moreover, DenseNet's feature map is much larger than ResNet's. As a result, the calculated convolution process is much longer than that of ResNet, so the training speed is slower than that of ResNet. The EfficientNet network has the best performance because of its balance between the depth and width and the input image size of the convolutional network achieved by setting certain parameter values. Most automatic image diagnoses made by basic artificial intelligence is based on cuboids or cubes on CT. In this study, the volume is reduced in the first segmentation, which greatly reduces the frequency of invalid information inputting, reduces the training time of the model and improves the accuracy rate.

Our study has some limitations. First, cochlear typing was not classified in detai l because the classification of cochlear malformations in this study was unbalanced, with IP1 and IP2 accounting for 89.32%. Subsequent studies targeting the type of cochlear malformation may be of value. Second, the sample size of our study is small due to the single institutional study design.

## Conclusion

Deep learning modalities can be valuable for diagnosing cochlear malformations on temporal bone CT images. This technique may become a useful diagnostic support tool. The performance of deep learning modalities was comparable to that of clinical experts. These results implied promising prospects for the clinical application of AI in diagnosing cochlear malformations based on CT images. Future studies should extend this approach to multicenter studies.

## ORCID ID

Zhenhua Li: 0000-0003-3230-9208

Langtao Zhou: 0000-0002-4290-867X

Xiang Bin: 0000-0002-5858-9706

Bin Liu: 0009-0001-0025-7709

Anzhou Tang: 0000-0001-6852-7317

Songhua Tan: 0009-0002-3137-7241

## Authors’ contributions

All authors contributed to the study conception and design. Investigation and methodology were performed by Xiang bin, Bin liu, project administration was performed by Anzhou Tang and Songhua Tan. Data curation and Writing-review & editing was performed by Zhenhua Li and Langtao Zhou and all authors commented on previous versions of the manuscript. All authors read and approved the final manuscript.

## Funding

This study has received funding by grants from Natural Science Foundation of Hunan Province (nº 2023JJ60302).

## Ethical approval

Institutional Review Board approval was obtained by The First Affiliated Hospital of Guangxi Medical University Medical Ethics Committee with grant nº 2022-KY-E-(135).

## Data availability statement

The authors declare that all data are available in repository.

## Declaration of competing interest

The authors of this manuscript declare no relationships with any companies, whose products or services may be related to the subject matter of the article.
